# Endoscopic-Assisted Scleral Fixated IOL in the Management of Secondary Aphakia in Children

**DOI:** 10.1155/2016/8501842

**Published:** 2016-08-10

**Authors:** Heba A. El Gendy, Hossam Eldin Khalil, Hazem Effat Haroun, Mohamed Wagieh El Deeb

**Affiliations:** ^1^Ophthalmology Department, Cairo University, Giza, Egypt; ^2^Ophthalmology Department, Beni-Suef University, Beni-Suef, Egypt

## Abstract

*Purpose*. To evaluate the short-term postoperative outcomes in endoscopic-assisted sclera fixation intraocular lens (IOL) for the management of secondary aphakia in children.* Methods*. This is a prospective study, whereas 40 aphakic eyes with absence of a good capsular support were implanted by endoscopy-assisted sclera fixation technique.* Results*. No major intraoperative complications were recorded. All cases were followed up for 6 months. Only transient ocular hypertension occurred in 10 (25%) eyes. Lens decentration and/or tilting were clinically detected in 2 eyes (5%). Ultrasonic biomicroscopic (UBM) examination revealed lens tilting in 2 (5%) of the operated eyes, despite the proper haptics positioning in the ciliary sulcus. Postoperative vitreous hemorrhage was reported in 5 eyes (12.5%) in the early postoperative period and retinal detachment in one eye. A postoperative refractive astigmatism ranging from 0.75 D to 3.75 D (mean 1.7 D ± 0.79) was recorded, as compared to mean preoperative values of 2.00 D, with no statistically significant differences being recorded (*p* ≥ 0.05). An improvement of BCVA, 1-2 lines on Snellen chart at the end of the follow-up period, was detected in 23 eyes (57.5%) with a mean of 0.6 ± 0.08 SD, as compared to a preoperative mean values of 0.5 ± 0.07 SD (*p* ≥ 0.05).* Conclusion*. Using an endoscope for transscleral suturing of intraocular lenses in aphakic pediatric eyes might be considered as being an effective technique that can reduce surgical complications, especially postoperative lens decentration.

## 1. Introduction

Loss of a good capsular support is one of the intraoperative complications that may interfere with the decision of primary intraocular lens implantation at the time of surgery, whereas the decision of secondary implantation might be considered later aiming for proper optical correction of the resultant aniseikonic condition following unilateral postoperative aphakia.

Several alternatives for the surgical correction of aphakia have been suggested (i.e., a posterior chamber intraocular lens (PCIOL) placed in the ciliary sulcus, or preferably in the capsular bag if possible); however in the absence of a good capsular support, an anterior chamber IOL, an iris-fixated IOL, or a sutured PCIOL would be suggested [1].

Moreover, the introduction of sutureless (sclera tunnel) fixation of PCIOLs and glued assisted fixation has been proposed for the management of the problem of secondary aphakia in children with lack of a good capsular support [2, 3].

Both transscleral suture fixation of posterior chamber lenses (PCLs) or sutureless fixation techniques in the absence of capsular support do provide the placement of the IOL in the posterior compartment being more fit to the anatomical position of the crystalline lens providing a good visual rehabilitation with a minimal long-term alteration of the blood-aqueous barrier [4].

Considering the transscleral suture fixation, the suture has to penetrate exactly through the ciliary sulcus, and the PCL haptics have to be directed into the sulcus and secured there in order to provide a proper centration of the implanted IOL [5, 6].

Being a blind technique, the transscleral fixated IOL may have many limitations and can cause various intraoperative and postoperative complications, where direct visualization of the haptic placement in the sulcus and the needle entry in the sutured IOL technique are prerequisites to achieve good results [7].

Intraocular endoscopy has been used for observing directly the needle penetration site for implantation of transsclerally sutured PCIOLs, trimming of the vitreous at the site of penetration and pars plana to remove any bands of the vitreous under visualization, and for meticulous examination of the posterior segment and the periphery of the retina, that might decrease the possibility of postoperative detachment or hemorrhage.

The aim of the current study is to investigate the short-term outcomes of endoscopic guided transsclerally sutured PCIOLs in a prospective series of consecutive aphakic cases of candidates for secondary implantation, regarding the postoperative IOL centration.

## 2. Patients and Methods

The current study was done in accordance with the ethical standards in the Declaration of Helsinki 1964 [8].

In this prospective, consecutive, series of cases, 40 aphakic eyes of 33 patients (18 males and 15 females), aged from 4 to 18 years (mean: 11.3 ± 4.8 SD), were recruited from patients attending the outpatient clinics in Kasr El Aini and Beni-Suef faculty hospitals. A detailed history was reported from parents/guardians who accompanied the patients. A full preoperative comprehensive ophthalmological examination was performed in all cases. Eyes with a history of previous local diseases, retinal conditions, congenital ocular abnormalities, and/or considerable anterior segment disfigurement were excluded from the current study.

All cases were operated on by the same surgical team in the ophthalmology department (Kasr EL Aini Hospital, Cairo University and Beni-Suef University Hospital, and Beni-Suef University), between January 2012 and July 2015.

All the cases were implanted because of a secondary decision following the initial surgery, whereas an endoscopic-assisted sulcus fixated PCIOL through typical 3-port vitrectomy approach was the procedure of choice, ensuring a well controlled needle entry as well as proper haptic positioning in the sulcus.

All patients or guardians, for patients under the age of 18, were requested to sign a full informed consent, regarding the operative techniques and the possible complications as well as the acceptance of the follow-up regimen.

### 2.1. Operative Details

The surgical procedures were as follows:

1. The site of the scleral flaps for the suturing of the IOL was detected, whereas conjunctival and corneal scars were encountered in some cases that influence choosing the site to avoid working in scared tissues. Another factor that determined the site of the corneal incision was the preoperative astigmatic axis aiming at minimizing the wound-related postoperative astigmatism.
2. Partial-thickness limbus-based triangular scleral flaps, 3.0 mm high and 2.0 mm wide, were fashioned at the planned sites.
3. 3 pars plana sclerotomies were performed, for infusion cannula, introducing endoscope probe and vitrectomy probe.
4. Typical 3-port pars plana anterior vitrectomy was performed to avoid vitreous incarceration at the site of positioning the PCIOL haptics.
5. A straight needle carrying 10-0 polypropylene was used to penetrate one of the two scleral beds parallel to the iris, 1.5 mm posterior to the posterior surgical limbus and holding it from the other site using 23-gauge needle [9, 10], whereas the exact needle penetration site was precisely observed intraocularly by insertion of the endoscope through the 2 o'clock positioned vitreous side port.
6. Examination of the retinal periphery and trimming of the anterior vitreous especially at the desired sites of fixation and detection of any complication at the penetration site that is, bleeding, vitreous band, or vitreous entangling with the haptics, were performed aided by the endoscope in all eyes.
7. A 7 mm 2-planned corneoscleral incision was created, with a 10-0 polypropylene suture loop to be withdrawn out of the eye through the opened wound. The loop was cut and tied to the haptics of the IOL.
8. The IOL was inserted through the corneal scleral wound and fixated to the ciliary sulcus by tying polypropylene suture to the scleral bed, with fixation of the PCIOL to the ciliary sulcus confirmed with the endoscope.
9. A specially designed rigid PMMA (polymethyl methacrylate) PCIOL with eyelet near the tip of the haptics to facilitate threading of the IOL, with the optic diameter of 6.5 mm and the overall length of 13.5 mm, was implanted in all eyes.
10. The sites of haptic fixation were checked after the sutures have been secured for any vitreous entrapment and/or bleeding to deal with at the end of the procedure.
11. A peripheral iridectomy was done routinely in all eyes before closing the corneal wound.
12. The 3-port sclerotomies were sutured using 8/0 vicryl sutures and the corneal wound was closed using 10/0 interrupted silk sutures
13. Subconjunctival Betamethasone (Diprofos) injection was injected routinely by the end of the procedure.

*Postoperative Follow-Up*. All cases were followed up for a period of 6 months postoperatively, whereas topical steroids and antibiotic were routinely continued for four weeks postoperatively in all cases.

Ocular reaction and the intraocular pressure were monitored in all eyes. The centration and tilting of the implanted PCIOL were checked clinically in all eyes using the slit lamp in which intraocular lens decentration was documented wherever; the center of the optic did not exist on the center of the cornea as measured from vertical and horizontal limbus to limbus distance by more than 1.0 mm. Moreover, the IOL tilting was ascertained when a small degree of tilting of the optics toward the visual axis exists clinically during slit-lamp examination.

Ultrasound biomicroscopy (UBM) was performed for all eyes at the end of the follow-up period to assess the haptics position and tilt of the sclerally fixated IOL using OTI-Scan 3000 with the acoustic axial resolution of 0.050 mm, electronic axial resolution of 0.027 mm, and a lateral electronic resolution of 0.035 mm. The scanning field was 18.5 mm in width and 14 mm in depth with a scanning angle of 34° that allows for a real time panoramic view to image the full anterior segment from sulcus to sulcus, the ciliary body, and peripheral retina that allows for the acoustic evaluation of both techniques.

Based on UBM images, we defined tilt as the deviation of the lens from sulcus to sulcus straight line in vertical and horizontal positions. Radial and transverse scans were also done over the site of the haptics for accurate localization and detection of any complications especially tilting, vitreous bands, or tissue incarceration.

Fundus examination was routinely performed at the immediate postoperative period and during periodic follow-up visits to assess the vitreoretinal condition and detect any complications.

The postoperative refraction and best corrected visual acuity (BCVA) were reported for all eyes by the end of the follow-up period.

The postoperative follow-up was performed by a masked independent observer; that is, the observer did not know the method used for the implant.

All the data were collected and subjected to statistical analysis using the computer program SPSS (Statistical Package for the Social Science, SPSS Inc., Chicago, IL, USA). The data were statistically described in terms of mean ± standard deviation (±SD), median and range, or frequencies and percentages when appropriate. Comparison of numerical data was done using Mann-Whitney *U* test. A *p* value less 0.05 was considered statistically significant.

## 3. Results

In the current study, 40 aphakic eyes (33 patients), that is, 18 males and 15 females, aged from 4 to 18 years (mean 11.3 ± 4.8 SD), were recruited from either patients attending the outpatient clinics, as a part of their routine postoperative follow-up regimens, or those who seek optical rehabilitation for their aphakia.

14 eyes (35%) gave a history of congenital cataract surgery before the age of 2 years, whereas 20 eyes (50%) presented with a history of trauma with injury to the crystalline lens that was removed while dealing with the traumatized eyes and 6 eyes (15%) gave a history of aphakia following complicated cataract surgery.

The preoperative BCVA ranged from 0.4 to 0.8 (mean 0.6 ± 0.11 SD), whereas the preoperative refractive astigmatism at the spectacle plane ranged from 0.5 D to 4.00 D (mean 2.03 D ± 0.98 D).

No major intraoperative complications were reported in any of the operated eyes; however intraoperative bleeding at the site of needle penetration was reported in 10 eyes (25%), which was managed intraoperatively by the endoscopic-guided vitrector and intraocular air injection.

Vitreous bands entangling the IOL haptics were detected in 5 eyes (12.5%) that were trimmed intraoperatively under direct visualization.

Postoperative mild to moderate anterior segment reaction, that is, flare + and ++ and no cells, was encountered in all cases that respond to the routine regimen of combined topical steroids and antibiotics, whereas pigment dispersion was reported in 6 eyes (15%).

Ocular hypertension (intraocular pressure greater than 21 mm Hg) was encountered in 10 eyes (25%) at the early postoperative period, that is, 1st 2 weeks, that was transient and well controlled at the final examination in all cases.

A mild to moderate vitreous hemorrhage occurred postoperatively in 5 eyes (12.5%), which was transient and absorbed within 1-2 weeks and required no additional surgical intervention.

Cystoid macular edema defined as clinically detected edema involving the macular area or lying within 500 *μ*m of the fovea was seen early postoperatively in 7 eyes (17.5%), in which moderate postoperative uveitis was encountered at the immediate postoperative period, which was improving throughout the follow-up visits.

In the current study, intraocular lens decentration was considered whereas the center of the optic did not exist on the center of the cornea as measured from vertical and horizontal limbus to limbus distance by more than 1.0 mm. Moreover, tilting of the IOL was considered when the IOL optic showed a small degree of tilting toward the visual axis, where IOL decentration was reported clinically in only one eye (2.5%) by the slit lamp examination, while clinically detected IOL tilting was noted in another eye (2.5%).

**Figure 1:**
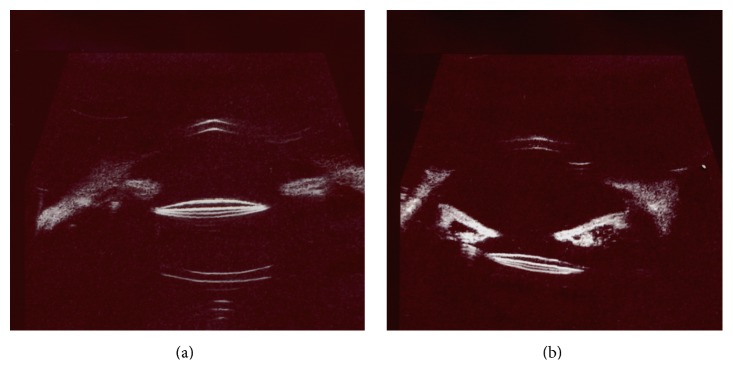
UBM scanning of postoperative lens position. (a) A well centered lens. (b) Lens tilting.

IOL tilt was further declared as the deviation of the lens from sulcus to sulcus in a straight line in vertical and horizontal positions using ultrasound biomicroscopy, whereas UBM examination revealed IOL tilting in 2 eyes (5%), with the IOL optics being displaced posteriorly (Figure 1).

Moreover, UBM guided evaluation of the haptic position of the implanted IOLs was implicated to report the haptics position in relation to the sulcus, that is, anterior or posterior to the sulcus, with all the implanted eyes showing well-positioned haptics acoustically using the UBM scan.

In addition, UBM visualization at the haptics sites revealed neither vitreous bands entrapped between the haptics and ciliary body at the insertion site nor tissue incarceration that is being managed intraoperatively.

A postoperative refractive astigmatism ranging from 0.75 D to 3.75 D (mean 1.7 D ± 0.79) was recorded by the end of the follow-up period, as compared to a mean preoperative values of 2.00 D, with no statistically significant differences being recorded (*p* ≥ 0.05).

An improvement of BCVA, that is, 1-2 lines on Snellen chart at the end of the follow-up period, was detected in 23 eyes (57.5%) with a mean of 0.6 ± 0.08 SD, as compared to preoperative mean values of 0.5 ± 0.07 SD (*p* ≥ 0.05).

Retinal detachment was noted during periodic follow-up, week 3 in one eye (2.5%) of a patient who gave a past history of blunt trauma, where the exact etiology of the detachment could not be declared clearly during the initial clinical examination, that is, no detected breaks. The patient was scheduled for vitrectomy where 2 peripheral holes were detected intraoperatively, away from the sites of haptic implantation, that were proposed to have developed during the step of vitreous shaving prior to implantation.

## 4. Discussion

The optical correction of aphakia in the pediatric age group has been considered as being a challenging situation, with the option of IOL implantation to be considered in the majority of cases.

In aphakic eyes with insufficient capsular support, scleral fixation of a PCIOL has been reported to be more superior than the anterior chamber IOL, being away from the corneal endothelium and being more anatomically placed; however, being a blind procedure with increasing the risks of intraoperative as well as postoperative complications made the procedure of ab externo scleral fixation PCIOLs in a rear position for many years [11].

Two technical difficulties have to be overcome in transscleral suture fixation of posterior chamber intraocular lenses (PCIOLs), especially in the pediatric eyes with previous anterior vitrectomy and decreased scleral rigidity: first, exact needle penetration through the sulcus and second, exact positioning of the PC IOL haptics in the ciliary sulcus. Incongruence of the two may lead to long-term complications by compression or even strangulation of ciliary processes [6, 12].

Various techniques of transscleral sulcus suturing of posterior chamber intraocular lenses (PCIOLs) have been described using ab interno [12, 13] or ab externo procedures aiming at reducing the rate of intra- and postoperative complications [14, 15].

In the current study an endoscopy-guided implantation of sclera-sutured IOL was applied aiming at decreasing the rate of intraoperative complications, as endoscopy-assisted technique may allow the proper intraoperative examination of the retinal periphery with subsequent dealing with any pathology, direct visualization of needle entry site, and intraoperative dealing with intraocular hemorrhages and/or vitreous entrapment.

IOL dislocation (IOL tilt or IOL decentration), a frequent complication of transscleral suture IOL implantation technique, has been reported at a rate of 28% in aphakic patients by Okada et al. [16].

In a retrospective comparative study, 22 eyes with transscleral fixated ab externo technique showed IOL dislocation at a rate of 23% as compared to 26 eyes with 0% dislocation rate with the endoscope-assisted fixation over a follow-up period of 3 months [15], which has been attributed to the improper positioning of the haptics in the ciliary sulcus, with the former technique.

According to the present study, we reported a clinical IOL displacement at a rate of 5% in the endoscope assisted eyes, ranging from either decentered or tilted IOL, that still seemed to be lower than the previously reported results regarding the ab externo technique [16].

However, the discrepancy between the reported endoscopic results [15] and ours regarding the IOL centration might be contributed to the selected age group in the present study with decreased scleral rigidity adding to the difficulty of the procedure.

Another factor that may affect the rate of IOL dislocation is the late degradation of the suture material [17]. In the current study, we use the 10/0 polypropylene sutures, although previous reports recommended the use of larger gauge sutures, that is, 9/0 polypropylene or 8/0 GoreTex. Unfortunately, this was not possibly available, which may add to the limitation of the current study; however, a longer follow-up is recommended for addressing late dislocation [18].

Ultrasound biomicroscopy, a technique that allows for a real time panoramic view to image the anterior segment, revealing the ciliary body and peripheral retina, was found as an adjuvant tool to evaluate the placement of the IOL postoperatively [19, 20].

Manabe et al. used ultrasound biomicroscopy to study eyes in which the IOL was sutured by the ab externo method and found that only 37% of the haptics were located adequately in the ciliary sulcus [21].

In another study by Alp et al., UBM examination showed that the rate of haptics located in the sulcus was statistically significantly higher in the endoilluminator-assisted group (64%) than in the control group (24%) (*p* = 0.001), which reflects the limitations of the blind needle penetration method for precise suturing in the ciliary sulcus [19].

In the current study, UBM examination revealed sulcus position of the haptics in all eyes, despite the clinically as well as the UBM recorded IOL displacement. Therefore, in a trial to explain that, we might attribute the cause of displacement to improper tying of the supporting sutures rather than lens haptic positioning.

Manabe et al. also demonstrated that 48% of the haptics in their sires were caught in the vitreous, even though anterior vitrectomy had been performed, that might induce vitreous traction and cause complications such as retinal detachment or macular hole and edema [21].

In the current study, UBM visualization at the haptics sites revealed no vitreous bands entrapped between the haptics and ciliary body at the insertion site, as needle penetration under direct vision by the aid of the endoscope aids the proper trimming of any vitreous bands at the haptic sites intraoperatively, as well as the proper examination of the retinal periphery, that might help in lowering the rates of retinal and macular complications.

In 2011, McAllister and Hirst reported ocular hypertention as the most common postoperative complication following sclera fixation, that is, 30.5% [11].

Again this was found to coincide with our results, although the postoperative ocular hypertension was recorded as being transient with IOP reaching its normal value by the end of the follow-up period.

Moreover, postoperative vitreous hemorrhage was previously reported at a rate of 16% following ab externo sclera fixation [12], whereas none of the presented eyes in the current study were reported with such a presentation due to the fact that using the endoscope intraoperatively added the privilege of the detection of intraoperative bleeding with the proper management accordingly.

Thus, in an attempt to overcome the high rate of problems that were reported with the conventional technique for scleral fixation of IOLs in the management of aphakia, we performed classic 3-port pars plana vitrectomy to remove as much vitreous gel as possible, with an endoscopic-assisted placement of the haptics precisely to the ciliary sulcus.

In the present study, the endoscope-assisted eyes showed favorable outcomes regarding the low incidence of IOL dislocation, with a favorable postoperative astigmatism.

The study thus suggests that endoscopic-assisted sclera fixation in children is a safe and effective technique that may reduce surgical complications in transscleral sulcus suturing of IOLs and helps the surgeon identify precisely the haptic positions, which may reduce the incidence of iris root and ciliary body damage.

## Supplementary Material

These techniques were not considered in the current study and were mentioned as a part of reviewing literure.
